# Bayesian evaluation of informative hypotheses in cluster-randomized trials

**DOI:** 10.3758/s13428-018-1149-x

**Published:** 2018-10-22

**Authors:** Mirjam Moerbeek

**Affiliations:** Department of Methodology and Statistics, Utrecht University, P.O. Box 80140, 3508 TC Utrecht, The Netherlands

**Keywords:** Cluster-randomized trial, Null hypothesis testing, Bayesian inference, Bayes factors, Informative hypotheses, Simulation study

## Abstract

Researchers often have informative hypotheses in mind when comparing means across treatment groups, such as *H*_1_ : *μ*_*A*_ < *μ*_*B*_ < *μ*_*C*_ and *H*_2_ : *μ*_*B*_ < *μ*_*A*_ < *μ*_*C*_, and want to compare these hypotheses to each other directly. This can be done by means of Bayesian inference. This article discusses the disadvantages of the frequentist approach to null hypothesis testing and the advantages of the Bayesian approach. It demonstrates how to use the Bayesian approach to hypothesis testing in the setting of cluster-randomized trials. The data from a school-based smoking prevention intervention with four treatment groups are used to illustrate the Bayesian approach. The main advantage of the Bayesian approach is that it provides a degree of evidence from the collected data in favor of an informative hypothesis. Furthermore, a simulation study was conducted to investigate how Bayes factors behave with cluster-randomized trials. The results from the simulation study showed that the Bayes factor increases with increasing number of clusters, cluster size, and effect size, and decreases with increasing intraclass correlation coefficient. The effect of the number of clusters is stronger than the effect of cluster size. With a small number of clusters, the effect of increasing cluster size may be weak, especially when the intraclass correlation coefficient is large. In conclusion, the study showed that the Bayes factor is affected by sample size and intraclass correlation similarly to how these parameters affect statistical power in the frequentist approach of null hypothesis significance testing. Bayesian evaluation may be used as an alternative to null hypotheses testing.

**Electronic supplementary material:**

The online version of this article (10.3758/s13428-018-1149-x) contains supplementary material, which is available to authorized users.

Over the past decades, the cluster-randomized trial has become a common research design in the behavioral, health, and medical sciences. With cluster-randomized trials, existing groups of subjects, such as schools, communities, or family practices, are randomized into two or more experimental conditions. Because subject outcomes within the same cluster are dependent, cluster-randomized trials are less efficient than individual randomized trials. Nevertheless, cluster randomization is often chosen in light of ethical and political realities, to reduce administrative and logistical costs, or to minimize the risk of contamination. For a more extensive introduction to this kind of trial, the reader is referred to Campbell and Walters (2014), Donner and Klar (2000), Eldridge and Kerry (2012), Hayes and Moulton (2009), and Murray (1998).

The aim of a cluster-randomized trial is to compare multiple treatment conditions to each other and to a control. Consider, as an example, two school-based smoking prevention interventions A and B, a control C, and a quantitative outcome variable (with higher scores being more favorable). The developer of intervention A may want to prove that his intervention is superior and that the control performs worst: *H*_1_ : *μ*_*C*_ < *μ*_*B*_ < *μ*_*A*_, where *μ* denotes the mean score. This is a so-called *informative hypothesis*, which gives an ordering of mean scores based on subjective beliefs, expectations, or findings in the literature. The developer of intervention B may have another informative hypothesis in mind: *H*_2_ : *μ*_*C*_ < *μ*_*A*_ < *μ*_*B*_. A school director who wants to implement an intervention may believe that both interventions perform better than the control but have no presumption which of the two performs better. The related hypothesis is *H*_3_ : (*μ*_*A*_, *μ*_*B*_) > *μ*_*C*_, where the notation (*μ*_*A*_, *μ*_*B*_) means there is no specific order of the two means. An unconstrained hypothesis does not impose constraints on the means, *H*_*a*_ : *μ*_*A*_, *μ*_*B*_, *μ*_*C*_; such a hypothesis may be formulated when there is no prior ordering of the effects of the two interventions and the control. This is also called the *encompassing hypothesis*, since all other hypotheses are nested within this one. For a more extensive introduction to Bayesian evaluation of informative hypotheses, see Hoijtink (2012).

The common practice when comparing the mean outcomes of *k* > 2 treatment conditions is to test the omnibus null hypothesis *H*_0_ : *μ*_1_ = *μ*_2_ = … = *μ*_*k*_, by means of a one-way analysis of variance (ANOVA). If this null hypothesis is rejected, post-hoc comparisons, accompanied by corrections for multiple hypothesis testing, can be performed in order to test which of the mean scores differ from each other. Here, *p* values are used to evaluate the significance of the effects. This approach of hypothesis testing is referred to as the *frequentist* approach.

The procedure of null hypothesis significance testing has been widely used in the empirical sciences for many decades, but it has also received severe criticism; see Klugkist, Van Wesel, and Bullens (2011) and Wagenmakers (2007) for a more elaborate discussion. First of all, the *p* value is generally used as a dichotomous decision rule in empirical research, such that effects are considered significant when the *p* value is less than *α* = .05. The value of the Type I error rate *α* should be chosen on the basis of the consequences of falsely rejecting the null hypothesis, but in practice the common *α* = .05 is often chosen without further justification. Using a fixed, one-dimensional “sacred” value *α* for distinguishing between significant and nonsignificant effects is not a shortcoming of the *p* value itself, which is a continuum on the interval [0, 1], but rather a problem of its current use in practice. Current null hypothesis significance testing practice “has become an anonymous amalgamation of the two incompatible procedures” of Fisher and Neyman/Pearson (Wagenmakers, 2007). This has resulted in the file-drawer effect, publication bias, questionable research practices, and even fraud (Simmons, Nelson, & Simonsohn, 2011).

Second, interpretation of the *p* value, which is the main result of null hypothesis significance testing, is not straightforward and is often misunderstood. The *p* value is the probability of obtaining the observed or even more extreme data, given that the null hypothesis is true: *P*(data | *H*_0_). However, researchers are more interested in the probability of the null hypothesis, given the data: *P*(*H*_0_ | data). In other words, they actually want to quantify the support for the null hypothesis given the data, rather than the support of the data given the null hypothesis. For a more extensive overview of misconceptions about how the *p* value should be interpreted, the reader is referred to Goodman (2008).

Third, researchers hardly ever believe the null hypothesis *H*_0_ : *μ*_1_ = *μ*_2_ = … = *μ*_*k*_ to be true (Cohen, 1990, 1994). It is unlikely that group means would be exactly equal to each other. In fact, researchers are more interested in informative hypotheses, such as the ones that were formulated earlier in this introduction. Researchers often want to compare these two hypotheses to each other directly, and the ANOVA procedure as described above cannot be used for this purpose.

Informative hypotheses *can* be directly compared to each other by using a Bayesian approach to hypothesis testing, without first having to perform an ANOVA.

The main concepts used in the Bayesian approach are the likelihood function of the data, as well as the prior and posterior distributions. The *prior distribution* represents knowledge with respect to the model parameters before collecting the data. Once the data are collected, their likelihood function is combined with the prior distribution to get the *posterior distribution*. Both prior and posterior are required in order to calculate a so-called *Bayes factor*, which is a quantification of the degree of evidence in the collected data *in favor of* an informative hypothesis (as compared to the unconstrained hypothesis *H*_*a*_). As such, the interpretation of the Bayes factor is more intuitive than that of the *p* value. Another strong advantage of the Bayes factor over the *p* value is that multiple hypotheses can be tested against each other simultaneously. Furthermore, the Bayes factor automatically takes the complexity (i.e., parsimony) of the inequality-constrained hypotheses into account. The Bayes factor may be considered a good alternative to the *p* value, but it should be used with care. Most of all, although the Bayes factor is a quantitative measure of evidence, it is likewise prone to being used in a dichotomous decision rule. A Bayes factor of > 1 implies more evidence for a given hypothesis than for the one to which the hypothesis is compared. If such a dichotomous decision rule were to become common research practice, the scientific community would end up mired in the same undesirable research practices (see the first point of criticism above on the *p* value) that it is currently trying to move away from.

Over the past decade, Bayesian evaluation of informative hypotheses has gained attention in the statistical literature, and tutorial articles have appeared in the social and behavioral science literature. Bayesian methods are suitable not only for observational studies, but also for randomized controlled trials. However, the application of Bayesian evaluation of informative hypotheses to cluster-randomized trials has been underexposed in the literature, while this trial design has become much more common in experimental research over the past decades.

The aim of the present contribution to the literature is to give a nontechnical introduction to Bayesian evaluation of informative hypotheses applied to cluster-randomized trials. The intended audience is empirical researchers who are involved in the design and analysis of cluster-randomized trials. The article aims to answer two research questions: (1) How effective are the interventions in a motivating example if estimates are done by the frequentist and Bayesian approaches, and how do these two approaches test the interventions against each other? (2) How is the Bayes factor related to sample size, effect size, and intraclass correlation coefficients?

The focus here is on inequality constraints, in which informative hypotheses are formulated on the basis of an ordering of means. In other words, equality constraints, in which two or more means are equal to each other, are not considered. Gu, Mulder, Deković, and Hoijtink (2014) argued that inequality-constrained hypotheses are a formal representation of a theory or expectation. As such, these hypotheses fulfill the requirement of constructing plausible, specific, and thus falsifiable hypotheses.

The contents of this contribution are as follows. A motivating example from smoking prevention is given in the next section, the mixed-effects statistical model is given, and informative hypotheses are formulated. The following section gives a short introduction to the Bayes factor for testing inequality-constrained hypotheses in cluster-randomized trials. The next section describes the design and results of a simulation study that was conducted to study the degree to which Bayes factors are influenced by the number of clusters, cluster size, intraclass correlation coefficient, and effect size. Thereafter, the example is continued, where frequentist and Bayesian approaches are used to analyze the data and test hypotheses. Conclusions and a discussion are given in the last section.

## Motivating example: School-based smoking prevention intervention

An example of a study based on cluster-randomized trials is a school-based smoking prevention intervention that was offered to elementary schoolchildren in the Netherlands (Ausems, Mesters, Van Breukelen, & De Vries, 2002). In this study, elementary schools were randomized to one of four conditions: a control, an in-school intervention (a school-based social influence program), an out-of-school intervention (three tailored letters with smoking prevention messages that were mailed to students’ homes), or both interventions.

The outcome variable that we consider here is the sum score on a scale that consists of 11 items measuring attitudes toward the disadvantages of smoking (range: 11–55). This variable was measured at pretest and posttest and will be treated as continuous in the analyses that follow. A higher score implied that a student was more negative toward the disadvantages of smoking. The 11 items are listed in the Appendix.

Responses from students in the same school cannot be considered independent, and such dependency has to be taken into account while analyzing these data. An appropriate model is the mixed-effects regression model (Hox, Moerbeek, & van de Schoot, 2018; Goldstein, 2011; Raudenbush & Bryk, 2002). The posttest score *y*_*ij*_, of student *i* in school *j*, is modeled as a function of the treatment condition and covariates:

1 $$ {y}_{ij}={\mu}_{CON}{d}_{CON,j}+{\mu}_{IN}{d}_{IN,j}+{\mu}_{OUT}{d}_{OUT,j}+{\mu}_{BOTH}{d}_{BOTH,j}+{\beta}_1{pretest}_{ij}+{\beta}_2{age}_{ij}+{u}_j+{e}_{ij}. $$

The means *μ* and dummy variables *d* have subscripts that refer to their treatment condition: *CON* = control = no intervention, *IN* = in-school intervention, *OUT* = out-of-school intervention, and *BOTH* = combination of both interventions. Two covariates are included in the model: pretest scores on attitude (range 15–55), and age (range 9.75–14 years). Both covariates are centered on their grand mean. The random terms *u*_*j*_~*N*(0, *τ*^2^), at the school level, and *e*_*ij*_~*N*(0, *σ*^2^), at the subject level, are assumed to be independent of each other. The intraclass correlation coefficient *ρ* = *τ*^2^/(*σ*^2^ + *τ*^2^) measures the proportion variance at the school level.

We now formulate informative hypotheses for this example. There are four treatment groups; hence, the number of orderings is 4 !  = 24. Not all orderings may be of interest, and a researcher may want to specify informative hypotheses based on his or her expectations or on findings in the literature. In the introduction of Ausems et al. (2002), they mentioned that “the combined approach was included because research indicates that multiple prevention strategies produce better results for the reduction of tobacco use.” Furthermore, one might expect that each of the interventions alone and the combined intervention would all do better than no intervention at all. One might formulate two different informative hypotheses based on different orderings of the in-school and out-of-school interventions:

2 $$ {\displaystyle \begin{array}{c}{H}_1:{\mu}_{CON}<{\mu}_{IN}<{\mu}_{OUT}<{\mu}_{BOTH},\\ {}{H}_2:{\mu}_{CON}<{\mu}_{OUT}<{\mu}_{IN}<{\mu}_{BOTH}\end{array}} $$

In both hypotheses, the control performs worst and the combined intervention performs best. In the first hypothesis, *H*_1_, the out-of-school intervention performs better than the in-school intervention, and in the second hypothesis, *H*_2_, the order of the two is reversed.

This set of hypotheses may not contain the true hypothesis. A straightforward solution to this problem would be to include the complement of the hypotheses of interest in the set of hypotheses to be tested (Böing-Messing, van Assen, Hofman, Hoijtink, & Mulder, 2017):

*H*_3_ : not(*H*_1_ *or H*_2_).

Since the four means can be ordered in 4 !  = 24 different ways, the complexity of hypotheses *H*_1_ and *H*_2_ is 1/24. Hypothesis 3 is the complement of hypotheses *H*_1_ and *H*_2_, and its complexity is therefore 1 − (2/24) = 22/24.

## Bayesian evaluation of informative hypotheses

Before the Bayes factor is defined, a short summary of Bayesian estimation is necessary. A general introduction to Bayesian estimation can be found in Gelman, Carlin, Stern, and Rubin (1995); an introduction to the Bayesian estimation of mixed-effect models is in section 2.13 of Goldstein (2011). The following concepts are of importance in Bayesian analysis: the likelihood function of the data, the prior distribution, and the posterior distribution. In what follows, the data are denoted *y* and the vector of the model parameters is denoted *θ*.

**Fig. 1:**
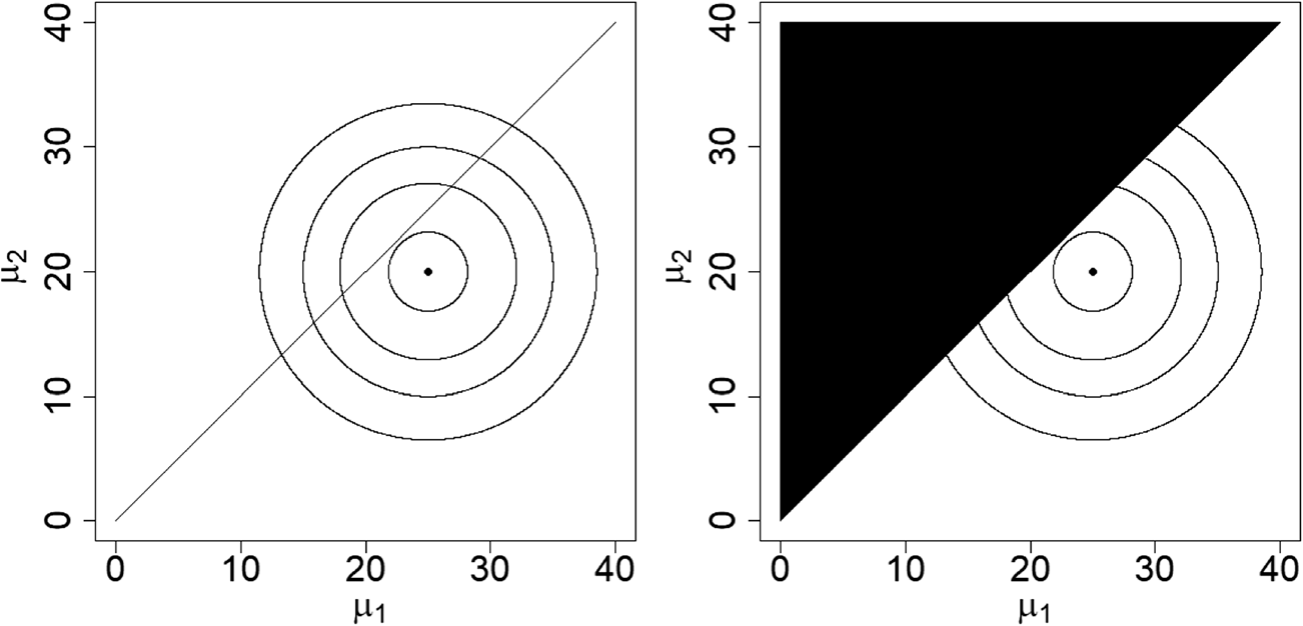
Two-dimensional representation of a likelihood function for two independent means *μ*_1_ and *μ*_2_ (left) and of the likelihood function for hypothesis *H*_*i*_ : *μ*_1_ > *μ*_2_ (right)

The likelihood function of the data, *f*(*y*| *θ*), can be interpreted as the support for the data *y* from each combination of the model parameters *θ*. The left panel of Fig. 1 gives a two-dimensional presentation of the likelihood function for two independent means *μ*_1_ and *μ*_2_, given some hypothetical data. The variance is ignored, since it does not fit within a two-dimensional figure that already includes both means. The circles represent combinations (*μ*_1_, *μ*_2_) that have the same likelihood value. The point in the middle, at *μ*_1_ = 25, *μ*_2_ = 20, represents the highest possible value of the likelihood given the data. The farther away from this point, the less likely is the combination (*μ*_1_, *μ*_2_). The point at *μ*_1_ = 25, *μ*_2_ = 20 is the so-called *maximum likelihood estimator*, which is the most common estimator in the frequentist approach.

In Bayesian estimation, not only the data but also prior information are used in estimation. The prior distribution *p*(*θ*) represents the a priori knowledge with respect to the model parameters *θ*—that is, the knowledge before the data *y* are actually observed. Such knowledge may be obtained from the literature or from an expert’s judgment. The software MLwiN uses inverse gamma priors for the variance components: *σ*^2^~inverse gamma(*a*_*e*0_, *b*_*e*0_) and *τ*^2^~inverse gamma(*a*_*u*0_, *b*_*u*0_). For the means, it uses normal priors: ${\beta}_i\sim N\left({\mu}_{i0},{\tau}_{i0}^2\right)$. Note that the parameters *a*_*e*0_, *b*_*e*0_, *a*_*u*0_, *b*_*u*0_, *μ*_*i*0_, and ${\tau}_{i0}^2$ are the so-called *hyperparameters* of the prior distributions.

The posterior distribution *p*(*θ*| *y*) combines the information with respect to the model parameters *θ* in the likelihood function of the data *y* and the prior distribution, on the basis of Bayes’s theorem:

3 $$ p\left(\theta\ |\ y\right)\propto f\left(y\ |\ \theta \right)\ p\left(\theta \right). $$

The posterior distribution is proportional to the product of the prior distribution and the likelihood function of the data. Only in simple cases can the posterior be derived analytically. In more complex cases, one needs to sample from the posterior using Markov chain Monte Carlo methods, such as Gibbs sampling. This is an iterative procedure in which each iteration produces a set of parameter values based on the parameter values of the previous iteration. The first iterations are usually discarded (this is called the *burn-in* phase), and inferences are based on the remainder iterations. Bayesian estimation for mixed-effect models can be performed in MLwiN (Rashbash, Steele, Browne, & Goldstein, 2015), WinBUGS (Lunn, Thomas, Best, & Spiegelhalter, 2000), and Mplus (Muthén & Muthén, 1998–2017). Once a large number of draws from the posterior have been made, the posterior can be summarized by statistics such as the median, the standard deviation, and the 95% credible interval, which is the interval bounded by the 2.5% and 97.5% quantiles.

Bayesian methods are suitable not only for the estimation of model parameters, but also for hypothesis testing. The Bayes factor of two informative hypotheses is defined as the ratio of their marginal likelihoods. The marginal likelihood of an informative hypothesis is the likelihood of the data conditional on the hypothesis, or the average of the likelihoods of the data over all parameter values that are in agreement with the hypothesis. The right panel of Fig. 1 shows the marginal likelihood for the informative hypothesis *H*_*i*_ : *μ*_1_ > *μ*_2_, calculated by integrating over the lower triangle (the upper triangle is indicated in black, meaning that it does not contribute to the marginal likelihood).

Marginal likelihoods most often are not easily calculated. Fortunately, the Bayes factor can be calculated without calculating the marginal likelihoods when the so-called *encompassing prior* approach is used (Klugkist & Hoijtink, 2007; Klugkist, Laudy, & Hoijtink, 2005). Klugkist and Hoijtink proposed two general guidelines for specification of the encompassing prior. First, this prior should not favor any of the informative hypotheses a priori. Such a prior is called a *neutral* prior (Hoijtink, 2012). For instance, if normal priors $N\left({\mu}_{i0},{\tau}_{i0}^2\right)$ are used for a set of means, then the same prior values for the hyperparameters *μ*_0_ and ${\tau}_0^2$ must be specified for each of these means. Second, the encompassing prior should be uninformative, meaning it is dominated by the data, so that the encompassing posterior is virtually independent of the prior. This is achieved by choosing a large value for the variance of the prior distribution of the means. The comparison of the hypotheses is then virtually objective.

For the encompassing prior approach, the Bayes factor of a hypothesis *H*_*i*_ versus the unconstrained hypothesis *H*_*a*_ : *μ*_1_, *μ*_2_, *μ*_3_, … is defined as

4 $$ {BF}_{ia}=\frac{f_i}{c_i}, $$

where *f*_*i*_ is the fit of hypothesis *H*_*i*_ and *c*_*i*_ is its complexity. The complexity *c*_*i*_ is the proportion of the encompassing prior distribution that is in agreement with the inequality-constrained hypothesis *H*_*i*_. The smaller this proportion, the more specific the hypothesis is. In other words, complexity is a measure of parsimoniousness and it is important to correct for complexity by including it in the denominator of the Bayes factor (4).

The fit of a hypothesis is the proportion of the posterior distribution that is in agreement with the inequality-constrained hypothesis *H*_*i*_. For each of the hypotheses, its fit can be calculated on the basis of a large number of draws from the Gibbs sampler. The end of this section shows how to calculate complexity and fit for a hypothetical example.

The Bayes factor *BF*_*ia*_ is a quantification of the degree of evidence in the data in favor of a hypothesis *H*_*i*_ against the unconstrained hypothesis *H*_*a*_. For example, *BF*_*ia*_ = 10 means that after observing the data, the support for *H*_*i*_ is ten times stronger than the support for *H*_*a*_. If *BF*_*ia*_ = 1, both hypotheses get equal support from the data; if *BF*_*ia*_ > 1, hypothesis *H*_*i*_ gets more support from the data, and if *BF*_*ia*_ < 1, hypothesis *H*_*a*_ gets more support from the data.

There are no general objective guidelines for interpretation of the value of a Bayes factor, just as the common significance level *α* = .05 is often chosen in null hypothesis significance testing without further justification (see the introduction). Table 1 shows proposed rules to interpret the strength of the evidence given by *BF*_*ia*_ (Kass & Raftery, 1995). It should be mentioned that these rules should not be used in a stringent manner. Some Bayesian statisticians even recommend not using such rules at all, but only reporting the value of *BF*_*ia*_, such that the reader can make his or her own judgment.

**Table 1:** Degree of evidence based on Bayes factors

Size of *BF*_*ia*_	Evidence in Favor of *H*_*i*_
1 to 3	not worth more than a bare mention
3 to 20	positive
20 to 150	strong
> 150	very strong

*BF*_*ia*_ is the Bayes factor of an inequality-constrained hypothesis *H*_*i*_ against the unconstrained hypothesis *H*_*a*_

The Bayes factor *BF*_*ii*′_, of a hypothesis *H*_*i*_ versus another hypothesis *H*_*i*′_, is defined as

5 $$ {BF}_{ii\prime }=\frac{BF_{ia}}{BF_{i\prime a}}. $$

The information in a set of Bayes factors may be presented in an alternative way, by using posterior model probabilities, which are defined as

6 $$ {PMP}_i=\frac{BF_{ia}}{1+{\sum}_i{BF}_{ia}} $$

for the informative hypotheses *H*_*i*_ (*i* = 1, …, *I*), and

7 $$ {PMP}_a=\frac{1}{1+{\sum}_i{BF}_{ia}} $$

for the unconstrained hypotheses *H*_*a*_. When *H*_*a*_ is not included in the set of hypotheses, then

8 $$ {PMP}_i=\frac{BF_{ia}}{\sum_i{BF}_{ia}}. $$

Posterior model probabilities allow for easier interpretation of the results when there are more than two hypotheses. They give the amount of support in the data for a given hypothesis within a set of hypotheses, on a scale that runs from 0 to 1. The posterior model probabilities are calculated from the Bayes factors, which in their turn are calculated under the assumption that the prior model probabilities are equal for each informative hypothesis.

### Computation of the Bayes factor

This subsection demonstrates how to compute the fit, complexity, and hence the Bayes factor, for the three hypotheses on two smoking prevention interventions and the control that were described in the introduction. It is inspired by Béland, Klugkist, Raîche, and Magis (2012).

The three hypotheses are *H*_1_ : *μ*_*A*_ < *μ*_*B*_ < *μ*_*C*_, *H*_2_ : *μ*_*B*_ < *μ*_*A*_ < *μ*_*C*_, and *H*_3_ : (*μ*_*A*_, *μ*_*B*_) < *μ*_*C*_. There are three means, which can be ordered in 3 !  = 6 unique ways. The complexity of hypotheses *H*_1_ and *H*_2_ is therefore *c*_1_ = *c*_2_ = 1/6. Hypothesis *H*_3_ is a combination of *H*_1_ and *H*_2_; hence, its complexity is *c*_3_ = 1/6 + 1/6 = 1/3. In other words, hypothesis *H*_3_ is less parsimonious than the other two hypotheses.

The fit of a hypothesis is the proportion of the posterior distribution that is in agreement with that hypothesis. Suppose 5,000 draws from the posterior have been taken using the Gibbs sampler. Table 2 gives hypothetical example draws for a handful of iterations. The drawn means *μ*_*A*_ = 6, *μ*_*B*_ = 4, and *μ*_*C*_ = 8 for the first iteration are in agreement with *H*_2_, and hence also with *H*_3_. This is recorded by the values of 1 in the second-to-last and last columns. Because these means are not in agreement with *H*_1_, the value 0 is recorded in the third-to-last column. The drawn means for the second iteration are in agreement with none of the three hypotheses; hence, a value of 0 is recorded in the last three columns. The drawn means for the third iteration are in agreement with both *H*_1_ and *H*_3_; hence, the value 1 appears in the last and third-to-last columns, and the value 0 appears in the second-to-last column. The reader can verify the results for Draws 4, 5, and 5,000.

**Table 2:** Draws from the Gibbs sampler for a hypothetical example

Iteration	*μ*_*A*_	*μ*_*B*_	*μ*_*C*_	*H*_1_	*H*_2_	*H*_3_
1	6	4	8	0	1	1
2	6	7	5	0	0	0
3	2	4	6	1	0	1
4	3	4	4	0	0	0
5	6	7	8	1	0	1
…						
5,000	6	5	8	0	1	1
Sum				2,145	789	2,934

The last line shows how often the posterior is in agreement with each of the three hypotheses. Note that the value for *H*_3_ is equal to the sum of the values for *H*_1_ and *H*_2_. The fit is expressed as a proportion: ${f}_1=\frac{\mathrm{2,145}}{\mathrm{5,000}}=.429$, ${f}_2=\frac{789}{\mathrm{5,000}}=.1578$, and ${f}_3=\frac{\mathrm{2,934}}{\mathrm{5,000}}=.5868$.

The Bayes factor for each hypothesis as compared to the unconstrained hypothesis *H*_*a*_ is now calculated as ${BF}_{1a}=\frac{f_1}{c_1}=\frac{0.429}{1/6}=2.574$; ${BF}_{2a}=\frac{f_2}{c_2}=\frac{0.1578}{1/6}=0.9468$ and ${BF}_{3a}=\frac{f_3}{c_3}=\frac{0.5868}{1/3}=1.7604$. Because *BF*_1*a*_ is highest, the support in the data is greatest for *H*_1_.

Bayes factors are more accurate when more draws from the posterior distribution are taken, but this results in a longer computation time. MLwiN reports diagnostics that can be used to judge whether enough draws have been taken (Browne, 2017).

## Simulation study

A simulation study was conducted to gain insight into the effects of cluster size, number of clusters, intraclass correlation coefficient, and effect size on Bayes factors in cluster-randomized trials. There were three treatments—*A*, *B*, and *C* (control)—and two informative hypotheses were considered:

9 $$ {\displaystyle \begin{array}{c}{H}_1:{\mu}_A<{\mu}_B<{\mu}_C,\\ {}{H}_2:{\mu}_B<{\mu}_A<{\mu}_C.\end{array}} $$

Table 3 represents the levels of the four factors considered in this simulation study: cluster size, number of clusters, intraclass correlation coefficient, and mean score per treatment condition. These levels cover a range of plausible values in cluster-randomized trials and result in a total of 72 combinations of levels. A cluster size of 5 is common for families, a cluster size of 10 is common for sports teams, a cluster size of 20 is common for school classes, and a cluster size of 40 represents larger clusters, such as departments in companies. The lowest number of clusters represents a cluster-randomized trial with a limited number of clusters; with the highest number of clusters, the model parameters and their standard errors are estimated without bias (Maas & Hox, 2005).

**Table 3:** Factors and their levels in the simulation study

Factor	Levels
Cluster size, *n*_1_	5, 10, 20, 40
Number of clusters, *n*_2_	30, 60, 90
Intraclass correlation coefficient, *ρ*	.025, .05, .1
Treatment means, (*μ*_*A*_, *μ*_*B*_, *μ*_*C*_)	(0, .2, .4), (0, .1, .2)

For each combination, 5,000 data sets were generated and analyzed by Bayesian estimation, with 10,000 iterations after a burn-in of 500 iterations. These results are based on the priors *τ*^2^~inverse gamma(0.001, 0.001) and *σ*^2^~inverse gamma(0.001, 0.001). For the fixed parameters, normal priors with mean zero and variance 1,000 were chosen. This large variance implies that the prior is uninformative; hence, the posterior distribution is almost entirely determined by the data. All data generation and analyses were done in R version 3.3.1 (Venables, Smith, & the R Core Team, 2016), using the Gibbs sampler given in Appendix 2.5 of Goldstein (2011). The annotated R syntax is presented in the supplementary material.

For each generated data set, the Bayes factor *BF*_12_ was calculated. Note here that because the data were generated under hypothesis *H*_1_, one would expect *BF*_12_ > 1 in many cases. For each combination of factor levels, the following criteria were calculated (Klaassen, Gu, & Hoijtink, 2015):

- Error probability: the proportion of data sets for which *BF*_12_ < 1. For these data sets there is more support in the data for *H*_2_ than for *H*_1_, which is erroneous, since the data were generated under hypothesis *H*_1_.
- Indecision probability: the proportion of data sets for which 1/3 < *BF*_12_ < 3. For these data sets, the evidence is not worth more than a bare mention (see Table 1).
- Median Bayes factor: Larger Bayes factors imply more support in the data for hypothesis *H*_1_.

**Fig. 2:**
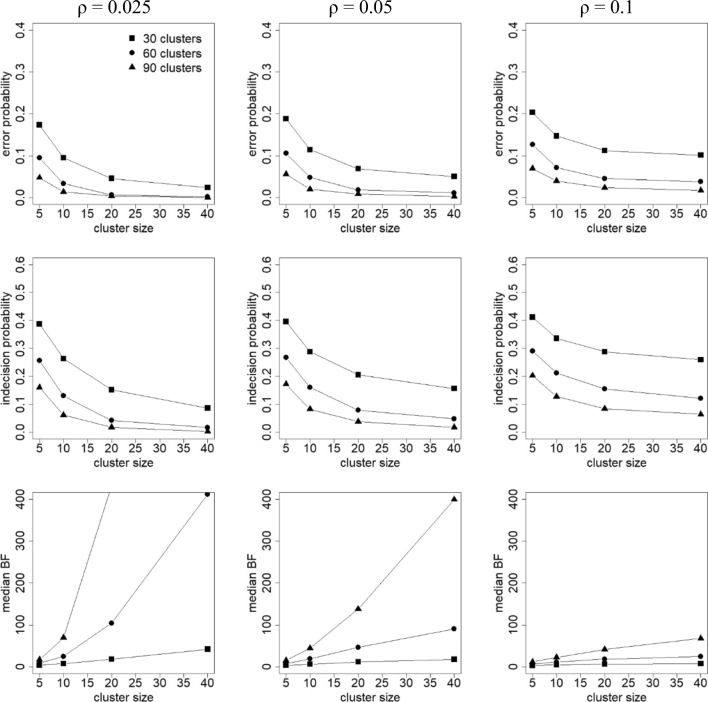
Error probability (top row), indecision probability (middle row), and median Bayes factor (BF; bottom row) as a function of intraclass correlation coefficient (columns), cluster size (horizontal axis within each plot), and number of clusters (separate lines within each plot). Population with (*μ*_*A*_, *μ*_*B*_, *μ*_*C*_) = (0, 0.2, 0.4). The legend as given in the upper left graph holds for all graphs

**Fig. 3:**
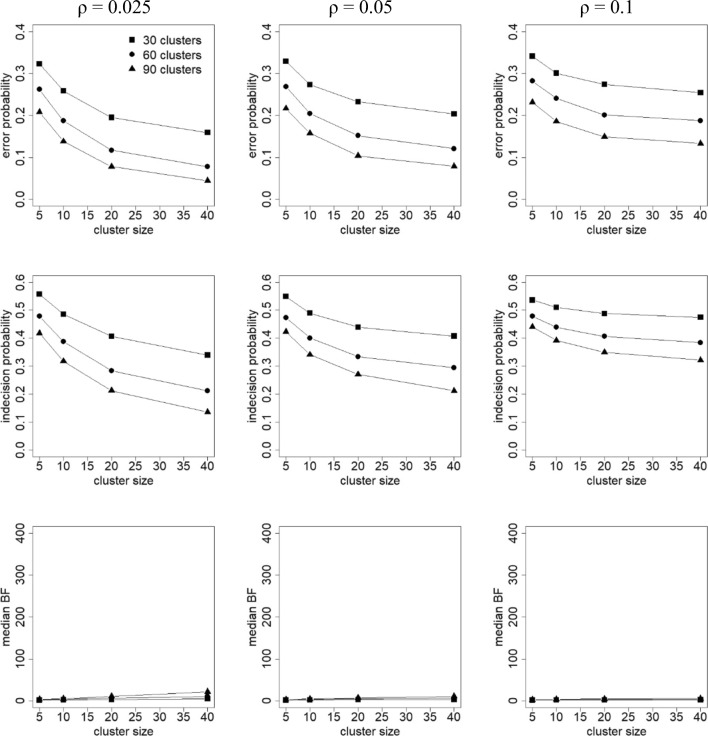
Error probability (top row), indecision probability (middle row), and median Bayes factor (BF; bottom row) as a function of intraclass correlation coefficient (columns), cluster size (horizontal axis within each plot), and number of clusters (separate lines within each plot). Population with (*μ*_*A*_, *μ*_*B*_, *μ*_*C*_) = (0, 0.1, 0.2). The legend given in the upper left graph holds for all graphs

Figure 2 shows results for the population with (*μ*_*A*_, *μ*_*B*_, *μ*_*C*_) = (0, .2, .4); the results for the population with (*μ*_*A*_, *μ*_*B*_, *μ*_*C*_) = (0, .1, .2) are very similar and are given in Fig. 3. Note that the lower left panel of Fig. 2 does not include the median BF for 90 clusters of size 40. This median BF has a value of 3,329, and inclusion of it in the graph would require the vertical axis to be scaled such that it would become difficult to distinguish and interpret the other results.

Figure 2 shows that the error probability and indecision probability decrease with increasing number of clusters and cluster size, and increase with increasing intraclass correlation coefficient. Reverse relationships hold for the median BF. The Bayes factor is consistent, which implies that the chance of selecting the true hypothesis increases with sample size. In general, the effect of the number of clusters is stronger than the effect of cluster size. The effect of increasing the cluster size becomes weaker when the intraclass correlation increases, especially when the number of clusters is small. Comparison of Figs. 2 and 3 shows that error/indecision probabilities become larger and median BFs become smaller when the effect size becomes smaller.

It should be mentioned that the findings from this simulation study are in line with the effects of the number of clusters, cluster size, intraclass correlation coefficient, and effect size on statistical power in the frequentist approach (Moerbeek & Teerenstra, 2016).

## Motivating example (continued)

At pretest the questionnaires were returned by 3,734 students within 143 schools; at posttest they were returned by 3,349 students within 140 schools. The main causes of attrition were absenteeism and difficulties with matching the pretest and posttest. The students with missing data on the posttest were excluded from the analysis. This has an effect of decreasing the power, but this should not be a problem, given the large sample size. Because the missing data are unlikely to be informative, excluding students with missing data at posttest should not bias the results.

The program MLwiN for multilevel analysis (Rasbash, Steele, Browne, & Goldstein, 2015) was used to fit model (1) to the data. The model explained 65% of the variance at the school level and 27% at the student level. All effects were significant at the *α* = .05 level, which is not surprising, given the large sample size. There was a positive relation between pretest and posttest attitudes (${\widehat{\beta}}_1=.563$), and students became less negative toward the disadvantages of smoking as they grew older (${\widehat{\beta}}_2=-.608$). On average, the students who received both interventions were most negative toward the disadvantages of smoking, and those in the control condition were least negative (${\widehat{\mu}}_{CON}=35.469,{\widehat{\mu}}_{IN}=38.293$, ${\widehat{\mu}}_{OUT}=39.122$, and ${\widehat{\mu}}_{BOTH}=39.428$).

A next step in the data analysis was to perform post-hoc tests to study which treatment groups differed from each other with respect to their mean outcomes, accompanied by a correction for multiple hypothesis testing. The mean outcome in the control condition significantly differed from the other three means (all *p*s < .001); no other significant differences were found (Bonferroni correction). The effect sizes, as expressed in Cohen’s *d*, were *d* = 0.41 (control vs. in-school), *d* = 0.53 (control vs. out-of-school), and *d* = 0.57 (control vs. both interventions), so the differences between the control and other conditions were medium in size. Due to the nonsignificant differences between the three treatment means, the frequentist approach does not answer the question of which of the two informative hypotheses in Eq. 2 was most supported by the data.

Table 4 summarizes the posterior distribution for each model parameter for the smoking prevention intervention. The posterior was obtained using Gibbs sampling in MLwiN version 2.36 (Rasbash et al., 2015), with 10,000 iterations after a burn-in of 500 iterations. These results are based on the priors *τ*^2^~inverse gamma(0.001, 0.001) and *σ*^2^~inverse gamma(0.001, 0.001). For the four treatment means, normal priors with mean 33 and variance 1,000,000 were used. The prior mean was the mean of the interval [11, 55] for the outcome measure. For the two covariates, priors with mean zero and variance 1,000,000 were used. This extremely large variance implies that the prior distributions were uninformative, meaning that the posterior distribution was almost entirely determined by the data. This implies that the selection of the best informative hypothesis was objective.

**Table 4:** Predictors of attitude toward the disadvantages of smoking

Variable	Median	Standard Deviation	95% Credible Interval
Mean effect control	35.487	0.412	(34.695, 36.321)
Mean effect in-school	38.304	0.411	(37.501, 39.109)
Mean effect out-of-school	39.109	0.399	(38.329, 39.891)
Mean effect both interventions	39.411	0.394	(38.644, 40.181)
Pretest attitude	0.564	0.016	(0.532, 0.596)
Age	– 0.606	0.218	(– 1.034, – 0.169)
Variance school level	3.576	0.716	(2.355, 5.134)
Variance student level	45.013	1.119	(42.894, 47.259)

Estimation by means of Bayesian estimation using uninformative priors. Both covariates are grand mean centered

The results showed the median of the posterior distribution, the posterior standard deviation, and the 95% credible interval, which is the interval bounded by the 2.5% and 97.5% quantiles of the posterior distribution. It should be noted that, since the priors were uninformative and the medians of the treatment means and the effects of covariates were almost entirely determined by the data, they were rather similar to the estimates from the frequentist approach. Remember that the frequentist approach does not include prior information.

The posterior distributions of the model parameters were exported from MLwiN to R (Venables et al., 2016), and a self-written syntax (available in the supplementary material) was used to calculate the Bayes factors and posterior model probabilities, shown in Table 5. The data show the highest support for hypothesis *H*_1_. This is not surprising, since the ordering of the means in this hypothesis is also found in the data (see Table 4). The Bayes factor for hypothesis *H*_3_, which is the complement of the other hypotheses, is lowest. This is explained by the fact that it has a high level of complexity combined with a low fit.

**Table 5:** Bayes factors and posterior model probabilities for the school-based smoking prevention intervention

Hypothesis	Complexity	Fit	*BF*_*ia*_	*PMP*_*i*_
*H*_1_ : *μ*_*CON*_ < *μ*_*IN*_ < *μ*_*OUT*_ < *μ*_*BOTH*_	1/24	.6351	15.242	.89
*H*_2_ : *μ*_*CON*_ < *μ*_*OUT*_ < *μ*_*IN*_ < *μ*_*BOTH*_	1/24	.0675	1.620	.09
*H*_3_ : complement of *H*_1_, *H*_2_	22/24	.2974	0.324	.02

*BF*_*ia*_, Bayes factor for *H*_*i*_ versus unconstrained hypothesis *H*_*α*_; *PMP*_*i*_, posterior model probability of *H*_*i*_

From the Bayes factors, we can draw the substantive conclusion that informative hypothesis *H*_1_ gets the highest support from the data. In other words, the control condition performs worst, the in-school intervention performs better, the out-of-school intervention performs even better, and the combination of both interventions performs best. We are not able to draw this conclusion on the basis of the frequentist approach.

## Conclusion and discussion

The frequentist and Bayesian approaches tested the hypotheses regarding the four experimental conditions in the empirical example in different ways. With the frequentist approach an omnibus test was performed, and it was concluded that the four conditions differed significantly in their means. Post-hoc tests revealed that the control differed significantly from the three interventions, whereas there were no significant differences among the interventions. The Bayesian approach of hypothesis testing allowed for a *direct* comparison of the various informative hypotheses of the four treatment means and provided a measure of the evidence for each of these hypotheses. The hypothesis *H*_1_ : *μ*_*CON*_ < *μ*_*IN*_ < *μ*_*OUT*_ < *μ*_*BOTH*_ received highest support from the data.

The results of the simulation study show how the Bayes factor is affected by the number of clusters, cluster size, effect size, and intraclass correlation coefficient. From those results, some general guidelines with respect to the design of a cluster-randomized trial can be drawn. First, it is more advantageous to increase the number of clusters than the cluster size. Second, increasing the cluster size has negligible effects when the intraclass correlation is large. Third, larger sample sizes are needed when effect sizes become smaller. Here it should be noted that these guidelines also hold when null hypothesis significance testing is used (Moerbeek & Teerenstra, 2016).

The results of the simulation study support the finding of a Bayes factor as high as 15.242 for informative hypothesis *H*_1_ in the example: The number of schools was rather large, and the ordering of the treatment means was as in *H*_1_. Had the number of schools been larger, an even larger Bayes factor might have been found. Increasing the number of pupils per school would have had a smaller impact. This implies that one should not worry too much if a few students per school drop out between pretest and posttest, as long as the missingness is not informative.

The Bayesian approach to hypothesis testing provides researchers a tool to evaluate informative hypotheses without first having to perform an omnibus test with an ANOVA. The additional advantage is that researchers are encouraged to think carefully about their hypotheses and the alternative competing hypotheses. They can discuss their expectations with colleagues and search the literature for results that are in conflict with their own expectations. This might have an advantageous effect on scientific progress.

This article is based on the encompassing prior approach developed by Klugkist, Laudy, and Hoijtink (2005) and Klugkist and Hoijtink (2007). An encompassing prior distribution should be chosen such that it is neutral: It does not favor any of the hypotheses a priori. This implies that the same prior means and variances should be used for all treatment means. Moreover, the variance should be large, so that the prior is uninformative. In the illustrative example, a prior mean equal to the mean of the range of the outcome variable was used. Another obvious choice is to use the mean in the control condition, which may be known from historical data or expert knowledge. However, the prior mean hardly influences the posterior (hence, the Bayes factors) if the prior variance is large. One advantage of Bayesian statistics that is often made use of by its proponents is the ability to incorporate informative prior information. That is true as long as the aim is to estimate model parameters. In such cases, unequal prior means and variances can be chosen for the treatment means and covariates. Recent research has developed methods to elicit prior information (Veen, Stoel, Zondervan-Zwijnenburg, & van de Schoot, 2017). However, if the aim is to evaluate informative hypotheses, the encompassing prior should be neutral and uninformative.

It should be noted here that the Bayesian approach is not the only approach to the evaluation of informative hypotheses. Another approach is based on an information criterion: the order-restricted information criterion (ORIC). See Kuiper and Hoijtink (2010) for an extensive overview of methods for the comparison of means: ANOVA, the ORIC, and Bayesian methods. An extension of the ORIC for multilevel data is currently in development.

It is important that the informative hypotheses be selected carefully, because only those hypotheses that are selected will be compared to each other. In the simulation study, it was expected that both smoking prevention interventions would have better effects than the control, resulting in a set of two informative hypotheses, *H*_1_ : *μ*_*A*_ < *μ*_*B*_ < *μ*_*C*_ and *H*_2_ : *μ*_*B*_ < *μ*_*A*_ < *μ*_*C*_. This set excludes the possibility of a hypothesis in which the control would do better than one or both interventions—for instance, the hypothesis *H*_3_ : *μ*_*C*_ < (*μ*_*A*_, *μ*_*B*_). If the data are indeed generated by hypothesis *H*_3_ but the focus is on the comparison of hypotheses *H*_1_ and *H*_2_ only, then a Bayesian evaluation of these two hypotheses will assign the highest Bayes factor to whichever of hypothesis *H*_1_ or *H*_2_ gets the most support from the data. This is often considered a drawback of Bayesian inference by its opponents, and it should be avoided through careful selection of plausible informative hypotheses. Alternatively, the complement of all informative hypotheses that are considered can be used, as was done in the example.

A great advantage of the Bayes factor is that it quantifies the strength of evidence for a hypothesis given the data, rather than the probability of the data given the null hypothesis. As such, it has a much more natural interpretation than the *p* value. Table 1 shows proposed rules to interpret the strength of evidence based on Bayes factors. It should be explicitly mentioned that such rules are always subjective, and (slightly) different values appear in other sources. At least, researchers should avoid using these rules in the same strict, unidimensional way the Type I error rate *α* is used, because this may again result in unwanted research practices, as was mentioned in the introduction. Some Bayesians even suggest reporting only the value of the Bayes factor, supported by median posterior estimates and credible intervals, and leaving the judgment to the reader. A similar argument has been made for null hypothesis significance testing (Cumming, 2011), that one should not rely only on the significance of effects as reflected by *p* values, but also focus on their relevance, as reflected by effect sizes and confidence intervals. Effect sizes are also important when the sample size of the study is large, so even small but irrelevant effects may become significant. In such cases, not only significance but also the relevance of the results should be discussed.

The Bayes factor is very useful in quantifying the results from an original study and its replication(s): One simply multiplies the Bayes factors obtained from each study in order to get a single measure of evidence based on multiple studies (Kuiper, Buskens, Raub, & Hoijtink, 2013). However, one should keep in mind that a large Bayes factor does not imply that a replication of the same study with the same sample size will again result in a large Bayes factor. This is also the case for *p* values in null hypothesis significance testing: A small *p* value does not give any guarantee that a replication of the study will also result in a small *p* value (Cumming, 2008). A replication of an initial study may thus result in nonsignificance, even if a significant effect was found in the initial study. This also implies that the sample size of a replication should not be determined on the basis of the *p* value of an initial study, but on an a priori power analysis (Atenafu, Hamid, Stephens, To, & Beyene, 2009).

A next step would be to provide tables for the relation between sample size and error probabilities, indecision probabilities, and median Bayes factors, similar to those for the relation between sample size and power for null hypothesis significance testing (Cohen, 1988). A first step has been made with simple randomized trials (i.e., without nesting of subjects within clusters; Klaassen et al., 2015), and an extension to cluster-randomized trials would be more than welcome.

### Author note

The author thanks Marlein Ausems and Hein de Vries for their approval to use the data from the Dutch smoking prevention intervention. This intervention is part of a European project, the Octopus Project, which is supported by financial grants from the European Commission (Soc 96 200568 05FO2/Soc 97 20249105F02). Herbert Hoijtink is acknowledged for his comments on the manuscript.

### Electronic supplementary material

ESM 1 (R 4.98 kb)

ESM 2 (R 1.09 kb)

## Appendix: List of the 11 items that measure attitudes toward the disadvantages of smoking

I will become nauseous I will cough I sense shortness of breath My eyes start to hurt Others inhale my smoke It is unwise of me It is expensive It is bad for my breath I annoy other people I will become addicted I regret having started

Each item is answered on 5-point scale, ranging from *positive* (1) to *very negative* (5) toward smoking.
